# Protein Kinase C Epsilon Promotes Cerebral Ischemic Tolerance Via Modulation of Mitochondrial Sirt5

**DOI:** 10.1038/srep29790

**Published:** 2016-07-20

**Authors:** Kahlilia C. Morris-Blanco, Kunjan R. Dave, Isabel Saul, Kevin B. Koronowski, Holly M. Stradecki, Miguel A. Perez-Pinzon

**Affiliations:** 1 Cerebral Vascular Disease Research Laboratories, University of Miami Miller School of Medicine, Miami, FL 33136, USA; 2 Neuroscience Program, University of Miami Miller School of Medicine, Miami, FL 33136, USA; 3 Department of Neurology, University of Miami Miller School of Medicine, Miami, FL 33136, USA

## Abstract

Sirtuin 5 (SIRT5) is a mitochondrial-localized NAD^+^-dependent lysine desuccinylase and a major regulator of the mitochondrial succinylome. We wanted to determine whether SIRT5 is activated by protein kinase C epsilon (PKCε)-mediated increases in mitochondrial Nampt and whether SIRT5 regulates mitochondrial bioenergetics and neuroprotection against cerebral ischemia. In isolated mitochondria from rat cortical cultures, PKCε activation increased SIRT5 levels and desuccinylation activity in a Nampt-dependent manner. PKCε activation did not lead to significant modifications in SIRT3 activity, the major mitochondrial lysine deacetylase. Assessments of mitochondrial bioenergetics in the cortex of wild type (WT) and SIRT5−/− mice revealed that SIRT5 regulates oxygen consumption in the presence of complex I, complex II, and complex IV substrates. To explore the potential role of SIRT5 in PKCε-mediated protection, we compared WT and SIRT5−/− mice by employing both *in vitro* and *in vivo* ischemia paradigms. PKCε-mediated decreases in cell death following oxygen-glucose deprivation were abolished in cortical cultures harvested from SIRT5−/− mice. Furthermore, PKCε failed to prevent cortical degeneration following MCAO in SIRT5−/− mice. Collectively this demonstrates that SIRT5 is an important mitochondrial enzyme for protection against metabolic and ischemic stress following PKCε activation in the brain.

Aberrant energy metabolism following cerebral ischemia/reperfusion induces mitochondrial impairments such as reduced respiration, free radical generation, and release of proapoptotic factors^1^,^2^. Consequently, mechanisms that maintain mitochondrial health provide crucial ischemic tolerance by enhancing neuronal viability following ischemic injury^3^. Our laboratory has previously shown that protein kinase C epsilon (PKCε), a serine/threonine kinase isoform of the PKC family, confers wide-scale mitochondrial protection and is an important signaling pathway in the induction of neuroprotection against otherwise lethal ischemic injury^4^,^5^,^6^,^7^.

PKCε activity influences a number of downstream signaling pathways that affect mitochondrial processes associated with ischemic neuroprotection. For example, cortical PKCε has been shown to regulate the serine/threonine kinase Akt, the mitogen-activated protein kinase/extracellular regulated kinase (MAPK) pathway, and AMP-activated protein kinase (AMPK), which are enzymes that modulate mitochondrial physiology by regulating transcription factors and gene expression^8^,^9^,^10^. In addition to regulating gene expression, PKCε can also directly regulate mitochondrial function. Studies in the hippocampus show that PKCε translocates to mitochondria where it phosphorylates the mitochondrial K^+^_ATP_ channel^6^, increases mitochondrial respiration, decreases mitochondrial ROS production, and inhibits cytochrome c release^7^ which collectively function to protect mitochondria against an ischemic insult.

The PKC family has been linked to sirtuins^11^,^12^, which are nicotinamide adenine dinucleotide (NAD^+^)-dependent lysine deacylases that are also associated with ischemic and mitochondrial neuroprotection^13^,^14^. There are seven mammalian sirtuin isoforms (SIRT1-7) which differ in their protein targets, subcellular localization, and enzymatic activity^15^. A major regulator of sirtuins is the enzyme, nicotinamide phosphoribosyl transferase (Nampt), which enhances sirtuin activity by increasing NAD^+^ levels^16^. Nampt is crucial in preventing neurodegeneration following cerebral ischemia as genetic studies show an exacerbation of injury when Nampt is knocked down^17^,^18^. Additionally, Nampt overexpression attenuated ischemic injury through activation of SIRT1^19^, indicating that the Nampt-sirtuin pathway may be important for ischemic protection. We recently demonstrated that PKCε enhances mitochondrial pools of Nampt and NAD^+^ in the cortex^9^. However, whether mitochondrial-localized sirtuins are regulated by PKCε-Nampt pathway or involved in ischemic neuroprotection has not been investigated.

SIRT3 and SIRT5 are localized to the mitochondria and have been identified as the major regulators of mitochondrial lysine deacetylation and lysine desuccinylation, respectively^20^,^21^. Proteomic analyses have revealed that about one-third of mitochondrial proteins contain lysine acetyl or lysine succinyl sites^20^,^21^, which indicates that SIRT3 and SIRT5 may have wide-ranging effects on mitochondrial function. The main goal of this study was to investigate whether PKCε is involved in regulating SIRT3 and SIRT5 and whether these sirtuins are involved in PKCε-mediated neuroprotection following cerebral ischemia.

## Results

### PKCε Plays a Critical Role in Ischemic Neuroprotective Pathways

**Figure 1:**
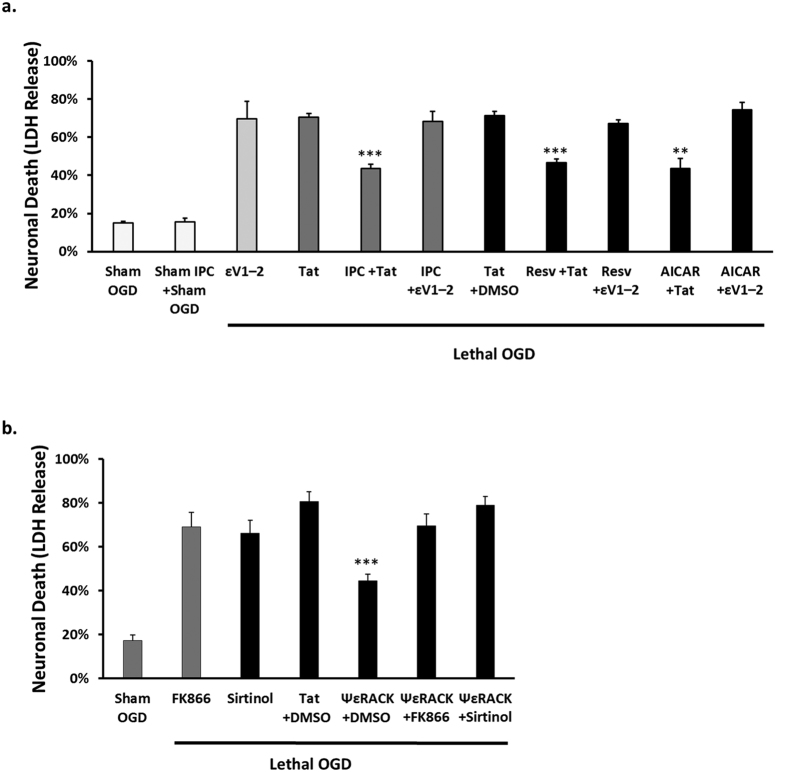
Nampt and Sirtuin Activity are Downstream of PKCε-Mediated Neuroprotection. Bar graphs representing neuronal cell death measured by LDH release at 48 hours of reperfusion after lethal OGD from rat neuronal-astrocyte cultures. (**a**) IPC, resveratrol (Resv) (25 μM), and the AMPK activator AICAR (0.5 mM)–mediated neuroprotection is blocked upon exposure to εV1–2 (100 nM), an inhibitor of PKCε activity. (**b**) ΨεRACK treatment provided significant protection against OGD-induced neuronal death which was blocked with the Nampt inhibitor FK866 (25 nM) or pan-sirtuin inhibitor sirtinol (10 μM). **p < 0.01, ***p < 0.001.

PKCε has an established role in preventing neurodegeneration following cerebral ischemia in both the cortex and hippocampus^5^,^6^,^22^. We have previously shown that activation of PKCε is required for the induction of neuronal survival following ischemic preconditioning (IPC)^10^,^23^, a paradigm where a brief sublethal ischemic insult protects the brain from a subsequent lethal ischemic injury. However, we never tested whether PKCε is a common pathway for the ischemic tolerance induced by other neuroprotective pathways that emulate IPC, such as resveratrol and AMPK^13^,^24^,^25^. To test the hypothesis that PKCε is a common pathway in IPC, resveratrol, and AMPK neuroprotection, rat neuronal-astrocyte cortical cultures were preconditioned by exposure for 1 hour to either oxygen-glucose deprivation (OGD) to induce IPC, resveratrol (25 μM), or the AMPK activator AICAR (0.5 mM), with or without the selective Tat-conjugated PKCε inhibitor, εV1–2 (100 nM). Forty-eight hours later, the cultures were subjected to 4 hours of lethal OGD and neuronal death was assessed with a lactate dehydrogenase (LDH) assay. This 48 hour time point was used to determine protection in the delayed window of preconditioning where protective effects are shown to be long lasting^26^. Sham treatment with the control peptide Tat resulted in 70.6% neuronal death whereas IPC showed significant protection with 44% neuronal death (p < 0.001, n = 5, Fig. 1a) following OGD. Resveratrol and AICAR treatment also showed significant protection with 47% (p < 0.001, n = 6) and 44% (p < 0.01, n = 5) neuronal death, respectively, in comparison to the control (Tat + dimethyl sulfoxide (DMSO)) with 71% neuronal death. The neuroprotection mediated by IPC, resveratrol, and AMPK activation was reversed with inhibition of PKCε activity (Fig. 1a), indicating the critical role of PKCε in these preconditioning neuroprotective pathways.

### PKCε Requires Nampt and Sirtuin Activity for Neuroprotection against OGD

We have previously shown that PKCε enhances levels of Nampt^9^, the rate-limiting enzyme involved in the production of NAD^+^. Nampt has been shown to provide protection against ischemic injury^17^,^18^ and increase the activity of sirtuins, which are NAD^+^-dependent lysine deacylases^16^,^19^. Both Nampt and sirtuins are integral enzymes for IPC-mediated protection^13^,^27^. Therefore, we next determined whether Nampt or sirtuin activity contributes to PKCε-mediated neuroprotection against lethal OGD in rat neuronal-astrocyte cortical cultures by using the Nampt inhibitor FK866 or the pan-sirtuin inhibitor, sirtinol. The cultures were treated for 1 hour with the PKCε agonist, ΨεRACK (100 nM), and then subjected to lethal OGD 48 hours later. PKCε activation significantly reduced neuronal death in comparison to control (45% compared to 81%, p < 0.001, n = 8, Fig. 1b). However, this neuroprotection was blocked when the cultures were treated with FK866 (25 nM) or sirtinol (10 μM) (p < 0.001, n = 8), demonstrating that Nampt and sirtuins are downstream of PKCε-mediated ischemic neuroprotection.

### PKCε Increases SIRT5 Desuccinylase Activity in Mitochondria

**Figure 2:**
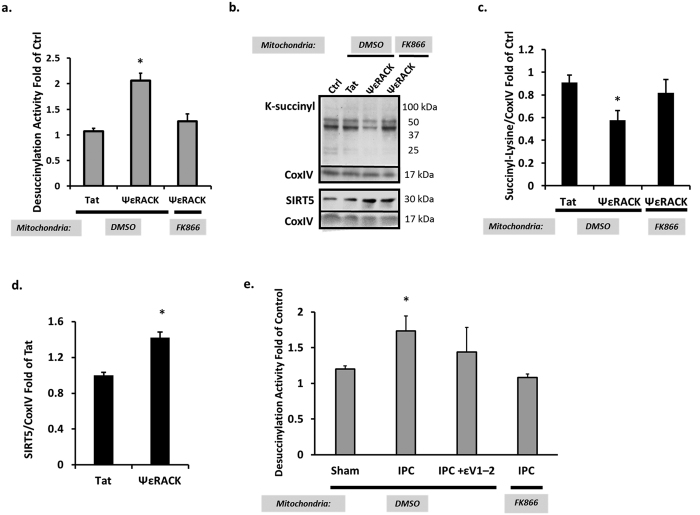
PKCε Increases SIRT5 Desuccinylase Activity via Mitochondrial Nampt. Mitochondria from rat neuronal-astrocyte cortical cultures isolated 48 hours following ΨεRACK (100 nM) treatment or IPC were exposed to DMSO, FK866 (Nampt inhibitor, 50 μM), or were not treated. (**a**) Increased desuccinylation activity following ΨεRACK treatment was blocked with mitochondrial Nampt inhibition. (**b–d**) Western blot analyses of lysine succinylation and SIRT5 in isolated cortical mitochondria. Gels were run under the same experimental conditions. Blots displayed are cropped and full-length blots are presented in Supplementary Figure S1. (**c**) PKCε activation decreased lysine succinylation which was reversed with exposure to FK866. (**d**) ΨεRACK treatment increased SIRT5 protein levels. (**e**) Increased desuccinylation activity following IPC was blocked by exposure to the PKCε inhibitor εV1–2 (100 nM) or FK866 in mitochondria. *p < 0.05.

Nampt has been shown to be a potent activator of sirtuin activity as sirtuins require NAD^+^ as a cofactor to drive their activity^16^,^19^. Since we previously showed that PKCε enhances mitochondrial Nampt and the mitochondrial NAD^+^/NADH ratio in the cortex^9^, we next wanted to determine whether PKCε regulates mitochondrial-localized sirtuin activity through Nampt activity. To test this, rat neuronal-astrocyte cortical cultures were exposed to ΨεRACK (100 nM) for one hour and the mitochondria were isolated 48 hours later. The mitochondrial fractions were treated for 45 minutes with DMSO vehicle control or the Nampt inhibitor FK866 (50 μM), which we have previously shown decreases mitochondrial pools of NAD^+ ^9. Mitochondrial sirtuin activity was analyzed by assessing the removal of a lysine-acetyl group or removal of a lysine-succinyl group from a substrate recognized by SIRT3 or SIRT5, respectively. ΨεRACK treatment resulted in a 2.1-fold increase in mitochondrial lysine desuccinylation activity (p < 0.05, n = 6) which was blocked when mitochondria were exposed to FK866 for 45 minutes (Fig. 2a). Western blot analysis confirmed decreased mitochondrial lysine succinylation 48 hours following ΨεRACK with a 34% reduction in lysine succinylation (p < 0.05, n = 5) which was reversed with the 45 minute FK866 exposure (Fig. 2b,c). ΨεRACK treatment also induced a 42% increase in SIRT5 levels in mitochondria (p < 0.05, Fig. 2d) which may contribute to the increased SIRT5 activity. However, since mitochondrial lysine succinylation was reduced by acute mitochondrial exposure to the Nampt inhibitor FK866, these results suggest that the increased SIRT5 activity is directly related to NAD^+^ availability provided by Nampt versus increased SIRT5 levels.

**Figure 3:**
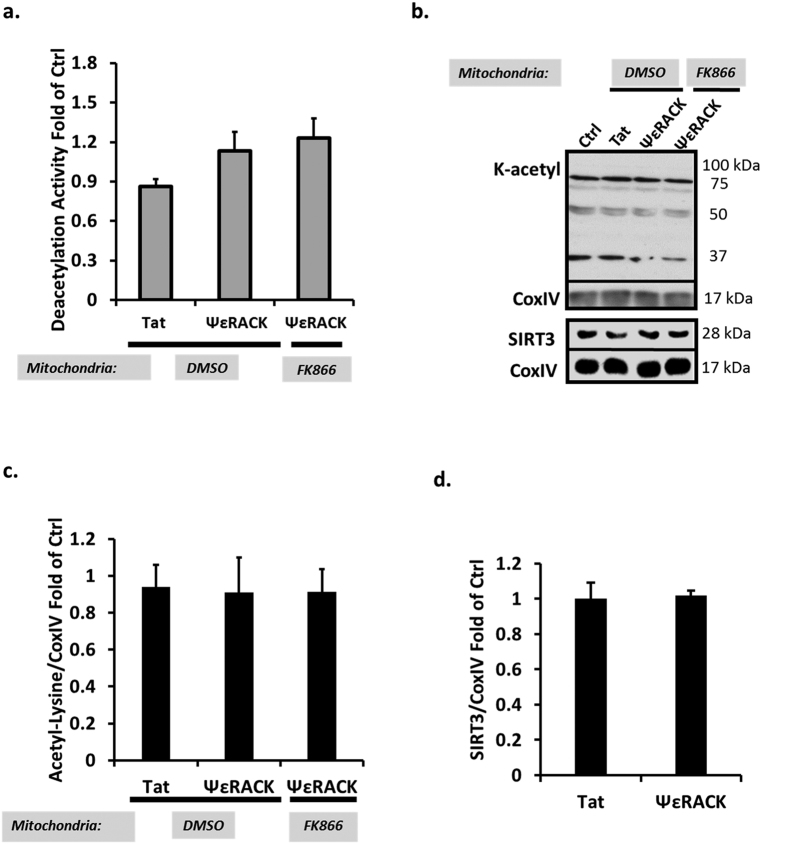
PKCε Does Not Enhance SIRT3 Deacetylase Activity. Mitochondria from rat neuronal-astrocyte cortical cultures isolated 48 hours following ΨεRACK treatment (100 nM) were exposed to DMSO, FK866 (Nampt inhibitor, 50 μM), or were not treated. (**a**) There were no differences in deacetylation activity following exposure to ΨεRACK. (**b–d**) Western blot analyses of lysine acetylation in isolated cortical mitochondria. Gels were run under the same experimental conditions. Blots displayed are cropped and full-length blots are presented in Supplementary Figure S1. (**c**) ΨεRACK treatment did not affect mitochondrial lysine acetylation. (**d**) SIRT3 levels remained the same following ΨεRACK treatment.

Since PKCε is a major downstream target in preconditioning paradigms, we next wanted to determine whether the PKCε-Nampt pathway is involved in regulating SIRT5 desuccinylase activity following IPC. Forty-eight hours following IPC, SIRT5 desuccinylase activity was increased in mitochondria from rat neuronal-astrocyte cultures (p < 0.05, n = 6). This increase in SIRT5 activity was abrogated by exposure to the PKCε inhibitor εV1–2 (100 nM) during IPC or FK866 (50 μM) to the mitochondria (Fig. 2e). In contrast to the SIRT5 activity results, ΨεRACK treatment had no effect on mitochondrial SIRT3 deacetylase activity (n = 5, Fig. 3a). No overall changes in the lysine-acetylation status of mitochondrial proteins or SIRT3 were observed 48 hours following ΨεRACK treatment (n = 5, Fig. 3b–d). Collectively, these data indicate that PKCε and mitochondrial Nampt are major regulators of SIRT5 desuccinylase activity, but not SIRT3 deacetylase activity, in cortical mitochondria.

**Figure 4:**
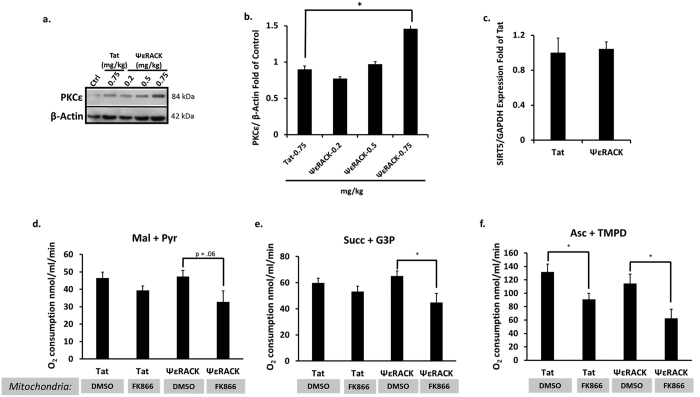
Nampt is Required for Maintenance of Mitochondrial Respiration Following PKCε Activation. (**a,b**) Western blot analysis of the particulate fraction from the WT mouse cortex 1 hour following intraperitoneal injection of ΨεRACK. Gels were run under the same experimental conditions. Blots displayed are cropped and full-length blots are presented in Supplementary Figure S1. (**b**) PKCε protein levels increased in the particulate fraction following a 0.75 mg/kg dose of ΨεRACK, indicating PKCε activation. (**c**) Real-time qPCR performed 24 hours following intraperitoneal injection of ΨεRACK showed no change in SIRT5 mRNA levels. (**d**–**f**) Mitochondria were isolated from mouse cortices and the rate of oxygen consumption was measured in the presence of complex I-linked substrates (malate (mal) and pyruvate (pyr)), complex II-linked substrates (succinate (succ) and G3P), and complex IV-linked substrates (ascorbate (asc) and TMPD) in the presence of ADP. Mitochondria from ΨεRACK-treated mice exposed to the Nampt inhibitor FK866 (50 μM) displayed reduced respiration in the presence of complex II and complex IV substrates compared to Tat-treated mice. *p < 0.05.

When PKCε is activated, it translocates to its isoform-specific receptors on intracellular membranes (found in the particulate fraction) that serve to localize PKCε to its intracellular substrates^28^. To determine the concentration of ΨεRACK required *in vivo* for PKCε activation in 129S1/SvImJ mice, we injected the mice with either the PKCε activator peptide, ΨεRACK (0.2, 0.5, or 0.75 mg/kg), or Tat control (0.75 mg/kg) i.p. and collected the particulate fraction from the cortex 1 hour later to determine translocation (activation) of PKCε. We found that 0.75 mg/kg of ΨεRACK induced a 50% increase in PKCε levels in the particulate fraction as compared to Tat injection (p < 0.05, n = 3, Fig. 4a,b), indicating PKCε activation. Since our previous experiment showed increased levels of SIRT5 in the mitochondria following PKCε activation, we next wanted to determine whether PKCε-mediated increases in mitochondrial SIRT5 levels were induced by increases in SIRT5 mRNA levels. We performed real-time qPCR on RNA isolated from mouse cortices 24 hours following intraperitoneal injection of Tat or ΨεRACK (0.75 mg/kg). ΨεRACK treatment had no effect on SIRT5 mRNA levels (n = 6, Fig. 4c), indicating PKCε activation may instead lead to increased stability of the SIRT5 protein in the mitochondria.

### SIRT5 Maintains Mitochondrial Respiration in the Cortex

Proteomic studies have shown that a large portion of lysine succinylated proteins in the mitochondria are involved in metabolic processes^20^,^29^. Therefore, we hypothesized that SIRT5 regulates mitochondrial respiration. As our previous experiment showed PKCε and mitochondrial Nampt are major regulators of SIRT5 activity in cortical mitochondria, we examined cortical mitochondrial respiration in mice following Tat or ΨεRACK treatment (0.75 mg/kg) and mitochondrial exposure to FK866 (50 μM) or DMSO control for 45 minutes. We have previously shown that PKCε activation increases mitochondrial NAD^+^ by nearly 60% which can be reversed when isolated mitochondria are exposed to FK866^9^. We tested oxygen consumption in isolated cortical mitochondria in the presence of substrates for complex I (pyruvate (5 mM) and malate (2.5 mM)), complex II (succinate (8 mM) and glycerol-3-phosphate (4 mM)), and complex IV (ascorbate (0.5 mM) and N,N,N′,N′-tetramethyl-p-phenylenediamine (TMPD, 200 mM)). Exposure to the Nampt inhibitor FK866 in the Tat controls reduced respiration rates in the presence of complex IV (p < 0.05, n = 7, Fig. 4f), demonstrating that mitochondrial Nampt may be important for basal mitochondrial activity. ΨεRACK treatment did not enhance mitochondrial respiration in basal conditions (Fig. 4d–f), but mitochondrial Nampt inhibition following ΨεRACK treatment reduced mitochondrial respiration in the presence of complex II and complex IV substrates (p < 0.05, n = 7, Fig. 4e,f). These data show that PKCε-dependent pathways may have a greater requirement for mitochondrial Nampt in maintaining mitochondrial respiration.

**Figure 5:**
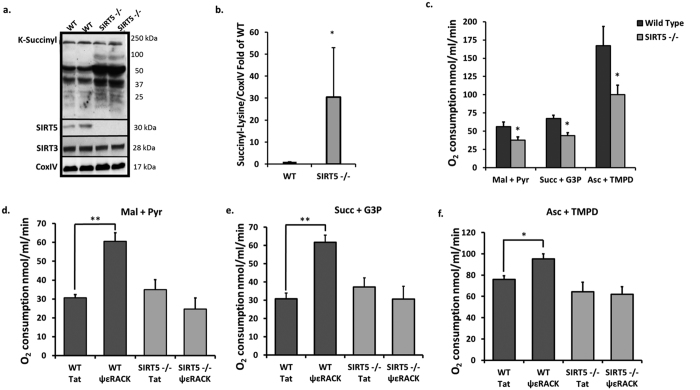
Absence of SIRT5 Reduces Cortical Mitochondrial Metabolism in Basal Conditions and Post-Ischemic Conditions. (**a,b**) Western blot analyses of lysine succinylation in isolated cortical mitochondria from WT or SIRT5−/− mice. Gels were run under the same experimental conditions. Blots displayed are cropped and full-length blots are presented in Supplementary Figure S1. (**b**) SIRT5−/− mice showed an increase in the overall lysine succinylation status of mitochondrial proteins from the cerebral cortex in comparison to WT. (**c–f**) Mitochondria were isolated from WT and SIRT5−/− mouse cortices and the rate of oxygen consumption was measured in the presence of complex I-linked substrates (5 mM malate (Mal) and 2.5 mM pyruvate (Pyr)), complex II-linked substrates (8 mM succinate (Succ) and 4 mM G3P), and complex IV-linked substrates (0.5 mM ascorbate (Asc) and 200 mM TMPD) in the presence of ADP. (**c**) Mitochondria from SIRT5−/− cortices displayed reduced respiration in the presence of complex I, complex II, and complex IV substrates. (**d–f**) WT or SIRT5−/− mice subjected to 85 min of MCAO 48 hours following injection with Tat or ΨεRACK. Mitochondria isolated 2 hours following MCAO show SIRT5 deficiency abrogates ΨεRACK-mediated increases in respiration. *p < 0.05, ** p < 0.01.

To directly assess whether SIRT5 is involved in regulating mitochondrial respiration in the cortex, we used SIRT5 knockout (SIRT5−/−) mice. SIRT5 has been identified as a lysine desuccinylase which widely regulates the mitochondrial succinylome^30^. Indeed, mitochondria isolated from cortical homogenates of SIRT5−/− mice showed significant increases in the lysine succinylation status of mitochondrial proteins in comparison to WT (Fig. 5a). Densitometric analysis revealed a 30-fold increase in overall succinylation in SIRT5−/− cortical mitochondria (p < 0.05, n = 3, Fig. 5b). This indicates that SIRT5 is a major regulator of mitochondrial protein succinylation in the cortex.

Cortical mitochondria from SIRT5−/− mice displayed significant reductions in oxygen consumption for each set of substrates as compared to WT mitochondria (n = 5, p < 0.05, Fig. 5c), indicating impairments in mitochondrial respiration at the basal level. Since our previous experiments showed that PKCε activates SIRT5 activity, we next wanted to determine whether SIRT5 is important for maintaining mitochondrial bioenergetics in ischemic conditions following PKCε activation. WT and SIRT5−/− mice were injected intraperitoneally with Tat or ΨεRACK (0.75 mg/kg) and then subjected to 85 minutes of middle cerebral artery occlusion (MCAO) 48 hours later. ΨεRACK treatment significantly enhanced mitochondrial respiration in the presence of substrates for complex I (p < 0.01), complex II (p < 0.01), and complex IV (p < 0.05) in WT mitochondria, but failed to do so in SIRT5 −/− mitochondria (n = 3–6, Fig. 5d–f). Collectively, these results show that SIRT5 activity is important for maintaining basal mitochondrial energy metabolism and required for PKCε-mediated mitochondrial protection following ischemic injury.

### PKCε Requires SIRT5 for Ischemic Neuroprotection

**Figure 6:**
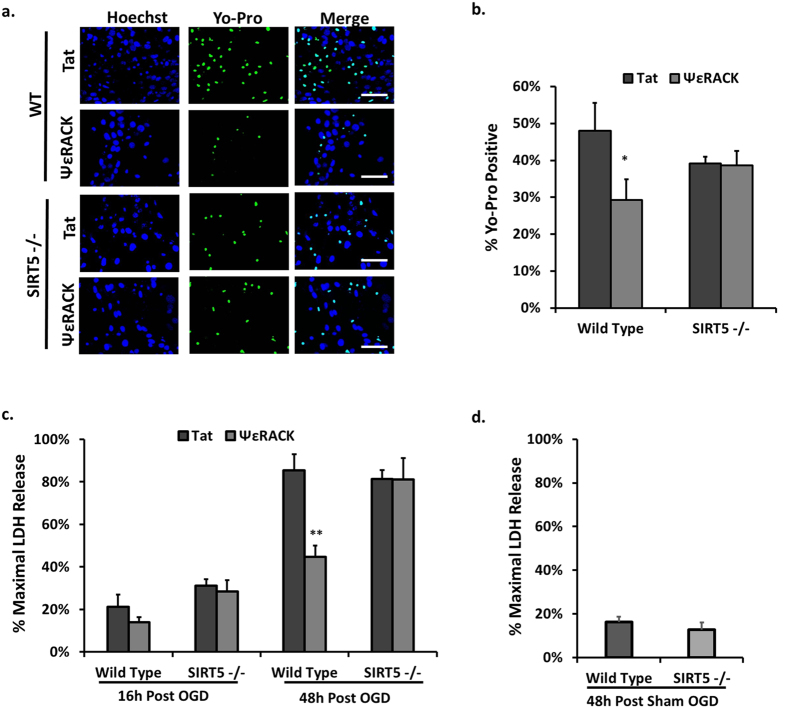
SIRT5 is Required for PKCε-mediated Protection Against OGD-induced Cell Death. (**a–d**) Neuronal-astrocyte cultures obtained from WT or SIRT5−/− mouse cortex. (**a**) Fluorescence images of cultures at 16 h of reperfusion after lethal OGD. Cultures were stained with the cell-permeable nuclear marker Hoechst (blue) and Yo-pro (green) which is permeable to apoptotic and necrotic cells. ΨεRACK treatment significantly decreased Yo-pro staining in WT cultures whereas ΨεRACK treatment in SIRT5−/− cultures had no protective effect; scale bar 100 μm. (**b**) Quantification of images in a. (**c,d**) Cell death measured by LDH release at 16 h and 48 h of reperfusion after OGD. (**c**) ΨεRACK treatment decreased LDH release at 48 h of reperfusion after lethal OGD in WT cultures, while SIRT5−/− cultures displayed no significant changes in LDH release. (**d**) LDH release in WT and SIRT5−/− 48 h following sham OGD treatment show no significant difference. * p < 0.05, **p < 0.01.

While nuclear sirtuins have been linked to ischemic neuroprotection^13^,^19^,^31^, a role for mitochondrial-localized sirtuins in mediating protection against cerebral ischemic injury has not yet been studied. Since the preceding experiments revealed that the PKCε-Nampt pathway selectively enhances mitochondrial SIRT5 activity and protein levels, we examined whether SIRT5 plays a role in ischemic protection mediated by PKCε. Neuronal-astrocyte cultures from WT or SIRT5−/− mice cortices were treated for one hour with ΨεRACK (100 nM) or Tat control (100 nM) 48 hours prior to a lethal OGD. We quantified the number of dead (necrotic) and dying (apoptotic) cells 16 hours following OGD by comparing the fluorescence of cells displaying Yo-pro (green) to the nuclear Hoeschst stain (blue) (Fig. 6a). Treatment with ΨεRACK reduced the number of Yo-pro positive cells in WT cultures (p < 0.05, n = 9), whereas this protection was abolished in SIRT5−/− cultures (n = 9, Fig. 6b). To further assess the effect of the PKCε-SIRT5 pathway on cell death, we also analyzed the amount of LDH released 16 and 48 hours following OGD. Unlike Yo-Pro staining which identifies both apoptotic and necrotic cells, the LDH assay only assesses necrotic cell death. In WT cultures, ΨεRACK treatment mediated a significant reduction in necrotic cell death 48 hours following OGD (p < 0.01, n = 7), whereas in SIRT5−/− cultures, ΨεRACK treatment failed to provide neuroprotection (n = 5, Fig. 6c). There was no difference in LDH release between WT and SIRT5−/− 48 hours following sham OGD (n = 6, Fig. 6d).

**Figure 7:**
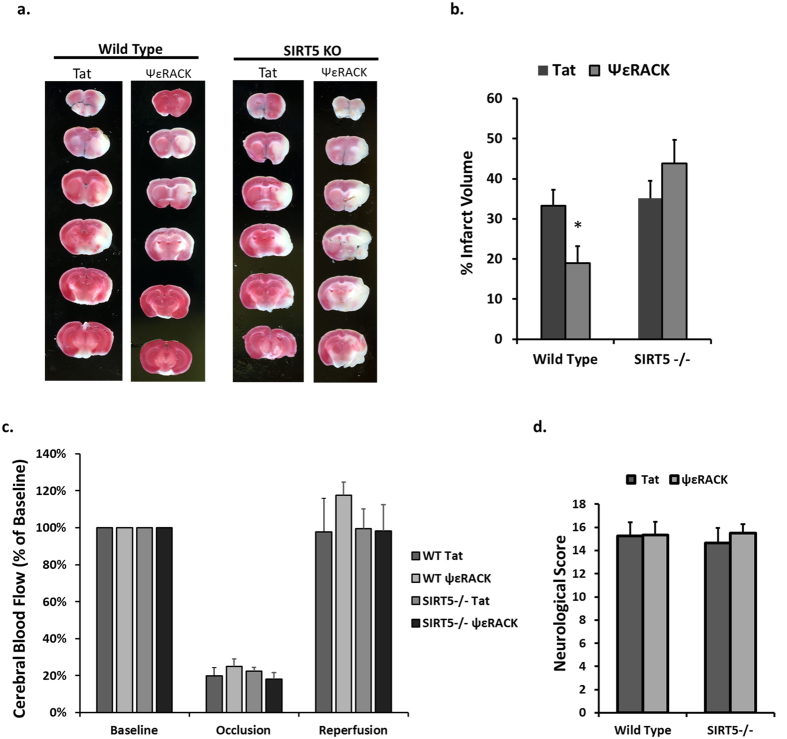
SIRT5 is Required for PKCε-Mediated Protection Against Cerebral Ischemia. WT and SIRT5−/− mice were injected with Tat or ΨεRACK intraperitoneally and 48 hours later were subjected to 85 minutes of MCAO. (**a**) Twenty-four hours following MCAO, TTC staining was used to analyze live versus dead tissue. (**b**) Quantification of the infarcted tissue 24 hours following MCAO showed loss of PKCε-mediated protection in SIRT5−/− mice cortices. (**c**) Laser Doppler analysis of cerebral blood flow during occlusion and reperfusion. (**d**) Neurological scores based on a neurobehavioral battery performed 24 hours following MCAO. *p < 0.05.

Our laboratory and others have previously shown that PKCε provides neuroprotection against ischemic injury in the rat cortex and hippocampus *in vivo*^5^,^22^. To determine whether PKCε-mediated ischemic neuroprotection is dependent on SIRT5 *in vivo*, we injected 0.75 mg/kg of Tat or ΨεRACK intraperitoneally in WT and SIRT5−/− mice then induced 85 minutes of MCAO 48 hours later. Twenty-four hours following MCAO, TTC staining was used to determine infarct size (Fig. 7a). ΨεRACK treatment reduced infarct size by 44% in comparison to Tat in WT mice (n = 9, p < 0.05; Fig. 7b). However, there was no difference between Tat (n = 10) and ΨεRACK (n = 8) treatment on infarct size following MCAO in SIRT5−/− mice, further confirming the integral role of SIRT5 in PKCε-mediated ischemic protection in the brain. Laser Doppler readings confirmed that the differences in infarct were not due to differences in cerebral blood flow during occlusion or reperfusion (Fig. 7c). No differences were observed in neurological scores based on a sensorimotor neurobehavioral battery (Fig. 7d) which indicates that despite the ability of PKCε to provide neuroprotection, its role in functional outcomes is not clear at this time.

## Discussion

The main goal of this study was to investigate whether PKCε regulates mitochondrial sirtuins and whether these sirtuins are involved in PKCε-mediated neuroprotection following cerebral ischemia. We report here that the PKCε-Nampt pathway is neuroprotective and enhances SIRT5 activity in cortical mitochondria. Our experiments revealed that SIRT5, the major mitochondrial lysine desuccinylase, is involved in regulating mitochondrial bioenergetics and neuroprotection against cerebral ischemia. We identified SIRT5 as a critical component of PKCε-induced protection against cell death following OGD *in vitro* and degeneration following MCAO *in vivo*. Our evidence further indicates that SIRT5 plays a major role in regulating mitochondrial respiration. This study provides the first evidence linking PKCε to SIRT5 activity as well as revealing a role for SIRT5 in mitochondrial bioenergetics and ischemic protection in the brain.

**Figure 8:**
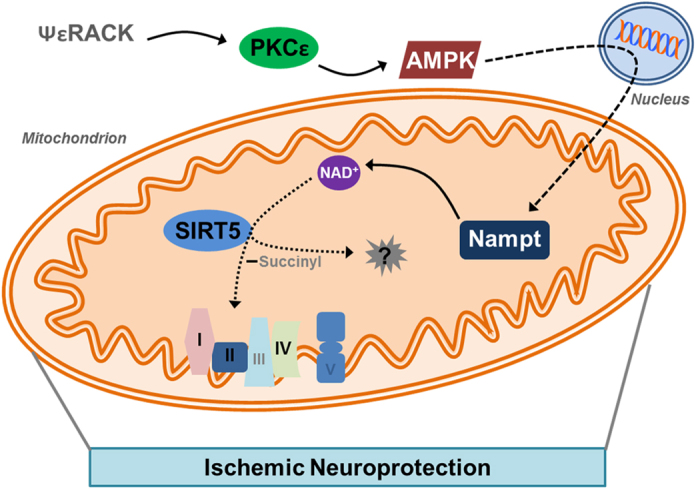
The PKCε-Nampt Pathway Provides Ischemic Neuroprotection via the Mitochondrial Desuccinylase SIRT5. Schematic diagram of the proposed model. PKCε is activated after treatment with ΨεRACK which enhances the activity of the transcriptional coactivator AMPK. AMPK upregulates the expression of Nampt which leads to increased Nampt localized to the mitochondria. Nampt enhances NAD^+^ levels which increases the lysine desuccinylase activity of SIRT5. SIRT5 desuccinylates multiple unknown targets in the mitochondria which lead to maintenance of mitochondrial complex activity and protection against an ischemic insult.

PKCε has an established role in providing protection against cerebral ischemia. Pharmacological activation of PKCε prevents neurodegeneration in both the cortex and hippocampus when administered prior, during, or following an ischemic insult^5^,^6^,^22^. PKCε has been shown to be critical for the robust neuroprotection provided by the IPC paradigm^10^,^23^, and we now show that PKCε may also be essential for the ischemic neuroprotection provided by resveratrol and the transcriptional co-activator AMPK. PKCε has been shown to prevent excitotoxic signaling and decrease microvascular blood flow during reperfusion^5^,^22^,^32^, two major pathologies that lead to mitochondrial dysfunction. PKCε further provides mitochondrial protection by regulating transcription factors and gene expression as well as directly phosphorylating mitochondrial proteins in a collective effort to enhance ATP production, decrease ROS production, maintain mitochondrial membrane potential, and reduce mitochondrial swelling^6^,^7^,^8^,^9^,^10^,^33^. We previously showed that PKCε activation of AMPK increases mitochondrial pools of Nampt^9^, an enzyme of primarily neuronal origin that enhances NAD^+^ and provides protection against cerebral ischemic injury^17^,^18^. Previous work has identified Nampt as a critical component of IPC-mediated protection^27^. In the current study, we show that Nampt is required for PKCε-mediated ischemic protection and regulation of SIRT5 activity, both of which represent additional pathways by which PKCε may regulate mitochondrial physiology and provide ischemic tolerance (Fig. 8). This pathway of protection most likely occurs in neurons since Nampt is primarily expressed in neurons^17^,^18^ and SIRT5 is more robustly expressed in neurons versus glial cells^34^.

The mammalian sirtuin family is made up of seven isoforms (SIRT1-7) which differ in their protein targets, subcellular localization, and enzymatic activity^15^. While nuclear or cytoplasmic sirtuins have been linked to ischemic protection in the brain^19^,^31^,^35^, the role of mitochondrial sirtuins in cerebral ischemia has not been fully addressed. For example, our laboratory^13^ and others^35^ have shown that sirtuins play an integral role in the ability of IPC to protect against ischemic stress, but the focus has always been on the nuclear or cytoplasmic activity of SIRT1. SIRT3-5 are the conventionally recognized mitochondrial sirtuins that each display unique enzymatic posttranslational modifications^36^. For example, SIRT3 is a lysine deacetylase, SIRT4 is a lysine ADP-ribosylase, and SIRT5 is a lysine desuccinylase^21^,^30^,^37^. There is also evidence that the SIRT1 deacetylase localizes to the mitochondria in addition to its cytoplasmic and nuclear localizations^38^,^39^. Unlike SIRT3 or SIRT5, SIRT1 does not regulate wide scale mitochondrial posttranslational modifications and only a few SIRT1 mitochondrial targets have been identified^38^,^39^. We previously showed that PKCε enhances mitochondrial pools of Nampt^9^, an enzyme involved in the production of NAD^+^ and a major regulator of sirtuin activity^16^. We also observed that PKCε activation led to a 60% increase in mitochondrial NAD^+^ levels in cortical neuronal-glial cultures, which could be blocked by acute exposure of the mitochondria to FK866, a Nampt enzyme inhibitor^9^. Since mitochondrial pools of NAD^+^ are distinct and regulated separately from the rest of the cell^40^,^41^, PKCε-mediated increases in mitochondrial Nampt represented a potential pathway involved in the regulation of mitochondrial-localized sirtuins. We were particularly interested in SIRT3 and SIRT5 which have been shown to regulate a significant portion of mitochondrial proteins through deacetylation or desuccinylation, respectively^20^,^21^.

Our current study indicates that the PKCε-Nampt pathway selectively enhances SIRT5 activity while SIRT3 activity remains unchanged. Although SIRT5 protein levels were increased, we showed that this was not due to enhanced expression of SIRT5. Therefore, it is likely that PKCε may improve the stability of the SIRT5 protein. Nevertheless, our experiments using the Nampt inhibitor on isolated mitochondria revealed that the increase in SIRT5 desuccinylase activity was directly related to increased NAD^+^ availability. Based on this evidence, Nampt (and the production of NAD^+^) appears to be the driving factor for increases in SIRT5 activity. However, the fact that both SIRT5 and Nampt^9^ levels are increased in the mitochondria following PKCε activation indicates that these enzymes can adequately work together to mediate their effects on mitochondrial function.

The fact that SIRT3 activity was not also enhanced was quite surprising due to the fact that all sirtuins rely on NAD^+^ to drive their enzymatic activity. A previous study performed by Yang *et al*.^40^ showed that enhanced mitochondrial NAD^+^ increased SIRT3 and SIRT4 activity in HEK293 cells^40^. Furthermore, SIRT3 and SIRT4 were critical to Nampt-mediated protection against genotoxic stress, while SIRT5 was not involved^40^. This evidence, in combination with the current study, indicates that SIRT5 may be modulated differently from the other mitochondrial sirtuins. SIRT1 has been shown to be regulated through posttranslational modifications such as sumolylation or phosphorylation^42^,^43^, but the mechanisms involved in the differential regulation of mitochondrial sirtuins are not clear at this time. However, the fact that all sirtuins are activated by increased NAD^+^ suggests that PKCε-mediated increases in SIRT5 activity may involve additional mechanisms that functionally cooperate with increased NAD^+^ availability.

Lysine succinylation was recently identified as a highly conserved posttranslational modification that induces significant structural changes and occurs with high abundance^29^. Further studies soon revealed that SIRT5 is a lysine desuccinylase^30^ and the major regulator of global mitochondrial succinylation^20^,^44^. Our experiments with SIRT5−/− mice confirmed that SIRT5 modulates wide-scale desuccinylation of mitochondrial proteins in the cortex. The fact that lysine succinylation sites are present on a large portion of mitochondrial proteins^44^ suggests that SIRT5 may be involved in regulating a number of mitochondrial processes. Proteomic analyses have revealed that many of the mitochondrial proteins that undergo lysine succinylation are involved in energy metabolism^20^,^44^. Indeed, our assessments of mitochondrial bioenergetics in the cortex of SIRT5−/− mice revealed that SIRT5 regulates basal oxygen consumption in the presence of complex I, complex II, and complex IV substrates. These data indicate the importance of SIRT5 activity in maintaining basal mitochondrial respiration. Interestingly, Nampt inhibition-alone in control mice only appeared to alter complex IV activity while Nampt was integral for PKCε-mediated maintenance of mitochondrial respiration indicating this enzyme is an important target for PKCε. Following ischemic injury, PKCε enhanced mitochondrial respiration in a SIRT5-dependent manner. Therefore, SIRT5 regulation of mitochondrial oxygen consumption may be a major contributing factor to the reduced PKCε-mediated neuroprotection we observed with SIRT5 deficiency.

The importance of studying SIRT5 in the brain is highlighted by the differences in SIRT5 function and protective abilities depending on cell-type. For example, SIRT5 regulates fatty oxidation pathways in MEFs and liver cells^20^ differently from the heart^45^. SIRT5 has been shown to both repress^20^ and increase^44^ mitochondrial enzymes. In L929 cells, SIRT5 was shown to promote cell death^46^, yet in cardiomyocytes and epithelial cells, SIRT5 prevents cell death^47^,^48^. In our previous study, we showed that PKCε increases AMPK activation in cortical neuronal-glial cells^9^, while AMPK overexpression in hepatocytes was shown to decrease SIRT5 expression^49^. However, since sirtuin activity is downstream to ischemic protective paradigms^13^,^35^, we hypothesized that SIRT5 would be involved in preventing ischemic degeneration in the brain.

We found that knockout of SIRT5 prevented PKCε-mediated protection against OGD-induced cell death in neuronal-astrocyte cultures and against MCAO injury in mice. In our study, SIRT5 inhibition alone did not lead to an increase in cell death following ischemic stress despite impaired mitochondrial bioenergetics. This mirrors previous findings that showed that sirtinol (pan-sirtuin inhibitor) treatment alone did not change the number of normal neurons nor exacerbate cell death in the hippocampus following asphyxial cardiac arrest, a model of global cerebral ischemia^14^. Our study indicates that the protective effects of SIRT5 against ischemic injury are downstream to PKCε activation and may not be inherent to the activity of the enzyme in basal conditions. We found that PKCε activation did not improve neurological scores 24 hours following MCAO. This may suggest that despite significant protection against cortical degeneration by PKCε, functional sensorimotor outcomes are not robust at early time points following MCAO. Although PKCε has widely been researched in cerebral ischemia, behavioral studies on the role of PKCε in experimental stroke models are lacking. Bright *et al*. 2013 showed that a mitochondrial-selective PKCε activator improved neurological scores 24 hours following MCAO^28^, which may suggest that mitochondrial PKCε activation provides better therapeutic potential. While functional outcome following PKCε activation is poorly understood and requires further research, our study provides strong evidence that SIRT5 is required for PKCε-mediated enhancements in mitochondrial bioenergetics as well as protection against MCAO-induced ischemic injury.

The exact mechanism by which SIRT5 prevents cell death is not clear at this time, but may include multiple mechanisms. For example, following cerebral ischemia, BAX translocates to the mitochondria where it binds to ATP/ADP translocases to form a mitochondrial permeability transition pore involved in triggering the release of cytochrome c and caspase-mediated apoptotic signaling cascades^50^. PKCε has been previously shown to provide protection to cardiomyocytes following ischemia by preventing apoptotic signaling^51^. Interestingly, SIRT5 interacts with and regulates levels of Bcl-xL, a protein that binds to BAX, in cardiomyocyte cultures^47^. Furthermore, knockdown of SIRT5 increases caspase 3/7 activity following oxidative stress^47^. SIRT5 has also been shown to interact with cytochrome c^52^, but the functional consequence of this interaction is not clear at this time. This evidence collectively indicates that SIRT5-mediated protection against cell death following PKCε activation may include several proteins and enzymes involved in apoptosis.

Many of the aberrant cellular processes following cerebral ischemia converge on the mitochondria resulting in energy metabolism failure, oxidative stress, and apoptotic signaling^2^. Over the past decade, PKCε has been identified as a major player involved in stabilizing mitochondria following ischemic injury^1^,^4^,^5^,^6^,^7^,^23^,^32^,^33^. As a wide-scale regulator of mitochondrial succinylation, SIRT5 may provide protection by regulating numerous mitochondrial proteins and enzymes as well as several mitochondrial processes. In this study, we show that SIRT5 maintains mitochondrial bioenergetics and protects against metabolic stressors and ischemic injury in the brain. These data represent the first report for the involvement of SIRT5, or any mitochondrial sirtuin, in ischemic neuroprotection. Further understanding how SIRT5 is preferentially activated as well as additional pathways by which SIRT5 mediates global mitochondrial protection, could help identify a novel therapeutic intervention against cerebral ischemia.

## Methods

All animal procedures were performed in accordance with the Guide for the Care and Use of Laboratory Animals published by the National Institutes of Health and approved by the Animal Care and Use Committee of the University of Miami.

Blinding was incorporated into the experimental design and methodology for all *in vivo* experiments; Double blinding was performed so that researchers were blind during administration of treatment, conduction of experiments, and analysis of data.

### Cortical Neuron and Astrocyte Cell Cultures

#### Astrocyte Cultures

Astrocyte cultures were prepared as previously described^53^ from Sprague–Dawley rats (Charles River Laboratories), or from wild type (WT) 129S1/SvImJ mice and SIRT5 homozygous knockout (SIRT5−/−) 129-Sirt5<tm1Fwa>/J mice (The Jackson Laboratory). P1-3 day old pups were anesthetized by isoflurane, sacrificed, and the brains were quickly removed. The cerebral cortices of the pups were isolated and the dissociated cells were plated at 1.5 cortical hemispheres/24-well plate with minimum essential medium (MEM; Life Technologies, Grand Island, NY) containing 10% fetal bovine serum (FBS), 10% equine serum, 2 mM glutamine, and 1% penicillin-streptomycin. After two weeks, the astrocytes were used as the source of the astrocyte monolayer for neuronal-astrocyte mixed cultures.

#### Rat Neuronal Cultures

Pregnant 18–19 day Sprague–Dawley rats were anesthetized by isoflurane and embryos were quickly removed and decapitated. The embryonic cerebral cortices were isolated and dissociated cortical cells were plated at 3 cortical hemispheres/24-well plate in MEM containing 2 mM glutamine and 5% FBS on the confluent monolayer of astrocytes previously prepared (neuronal-astrocyte cultures). Every 3–4 days, half of the media was changed with normal maintenance media consisting of MEM containing 2 mM glutamine.

#### Mouse Neuronal Cultures

Neuronal cultures were prepared from P0-P1 day old pups^54^ from WT or SIRT5−/− mice. Pups were anesthetized by isoflurane, sacrificed, and the brains quickly removed. Cerebral cortices were isolated and the dissociated cells were plated at 2 cortical hemispheres/24-well plate with MEM Eagle’s with Earle’s BSS (Life Technologies) supplemented with 10% heat-inactivated FBS, 1 mM sodium pyruvate, 2 mM glutamine, and penicillin/streptomycin (Life Technologies). After 4 hours *in vitro*, the plating media was removed and replaced with maintenance media composed of Neurobasal medium supplemented with B-27, 2 mM glutamine, and penicillin/streptomycin (Life Technologies). The neurons were plated on the previously prepared astrocytes described above to generate mixed neuronal-astrocyte cultures.

All cultures were kept in an incubator at 5% CO_2_ at 37 °C and used after two weeks *in vitro*.

### Pharmacological Treatments

For *in vitro* experiments, cultures were exposed for 1 hour to 25 μM resveratrol (Sigma, St. Louis, MO, USA), 100 nM of Tat-conjugated ΨεRACK (PKCε activator, KAI Pharmaceuticals, San Francisco, CA, USA), or 0.5 mM AICAR (AMPK activator, Sigma), with or without 25 nM FK866 (Nampt inhibitor, EMD Millipore, Billerica, MA, USA), 10 μM sirtinol (pan-sirtuin inhibitor), or 100 nM of Tat-conjugated εV1–2 (PKCε inhibitor, KAI Pharmaceuticals). Following the 1 hour pharmacological treatment, cultures were maintained in normal maintenance media and were used for experimental analyses 48 hours following pharmacological preconditioning treatment.

For mitochondria isolation experiments, the mitochondria were exposed to DMSO or 50 nM of FK866 for 45 minutes to reduce mitochondrial NAD^+^ levels as previously described^9^.

For *in vivo* experiments, WT or SIRT5−/− mice underwent intraperitoneal (i.p.) injection with 0.75 mg/kg (unless otherwise stated) of Tat-conjugated ΨεRACK or the Tat peptide. Cortices were collected for analysis 1 hour following injection for PKCε activation studies or mice were used for MCAO experiments (described below).

### Oxygen-Glucose Deprivation

To mimic ischemia *in vitro*, we subjected cultures to oxygen-glucose deprivation (OGD). Cultures were washed twice with glucose-free Hank’s balanced salt solution (pH 7.4) of the following constitution (in mM): 1.26 CaCl_2_·2H_2_O, 5.37 KCl, 0.44 KH_2_PO_4_, 0.49 MgCl_2_, 0.41 MgSO_4_·7H_2_O, 136.9 NaCl, 4.17 NaH-CO_3_, 0.34 Na_2_HPO_4_·7H_2_O, and 10 HEPES (Sigma). Cultures were then transferred to an anaerobic chamber (Coy Laboratory Products, Grass Lake, MI) gassed with 90% N_2_, 5% CO_2_, 5% H_2_ at 37 °C for 1 hour to induce ischemic preconditioning (IPC), 3 hours for lethal OGD in mice cultures, or 4 hours for lethal OGD in rat cultures, after which the media was replaced with normal maintenance media and placed back into the normoxic incubator (5% CO_2_ at 37 °C). For sham OGD, cultures were washed twice with HBSS containing glucose and placed into the normoxic incubator (5% CO_2_ at 37 °C) for the selected times, after which the media was replaced with normal maintenance media.

### Cell Death Measurements

#### Lactate Dehydrogenase Assay

To determine neuronal death, cytotoxicity was measured by lactate dehydrogenase (LDH) released into culture medium for 16 or 48 hours following lethal OGD using a Cytotoxicity Detection Kit (Roche Diagnostics Corporation, Indianapolis, IN, USA). Maximal neuronal LDH release was evoked by exposure to *N*-methyl-d-aspartate (NMDA) (500 μM), an excitotoxin that preferentially kills neurons, for 48 hours. Maximal total cell death was determined by exposure to 1% Triton for 10 minutes. LDH release was measured at an absorbance at 340 nm using a microplate reader (Molecular Devices, Sunnyvale, CA). Values were expressed relative to LDH measurement from maximal neuronal or maximal total LDH release.

#### Yo-Pro Assay

Mouse cortical cultures were grown onto glass coverslips in 24-well plates and cultured *in vitro* for two weeks as described above. Sixteen hours following OGD, cultures were exposed to the nuclear marker Hoechst 33342 (Life Technologies) and apoptosis marker Yo-Pro-1 (10 μM, Life Technologies) for 15 min at 37 °C to identify both dying (apoptotic) and dead cells. Coverslips were then placed into a petri dish containing normal maintenance media and two-photon microscopy controlled by Lasersharp 2000 software (BioRad) was used for live-cell imaging. The number of apoptotic and dead cells was quantified as the proportion of Yo-pro positive cells to the total number of cells identified by the Hoechst stain.

### Cellular Fractionation

#### Mitochondrial Fractionation

Cortices or cultures were washed twice in cold (4 °C) isolation medium consisting of 225 mM mannitol, 75 mM sucrose, 5 mM HEPES, and 1 mM EGTA, pH 7.4 and then homogenized in a hand-operated glass Teflon homogenizer in isolation medium. The homogenates were centrifuged at 1300 × *g* for 5 minutes. The resulting supernatant was centrifuged at 17,000 × *g* for 10 minutes. The pellet formed was used as the source of the crude mitochondrial fraction. For mitochondrial purification, the pellet was resuspended in 15% Percoll and layered over a preformed gradient of 22% Percoll which was layered over 50% Percoll^55^. The Percoll density gradient was centrifuged at 17,000 × *g* for 10 minutes and the purified mitochondria were collected at the interface between 50% and 22% gradients. The purified mitochondrial sample was centrifuged at 7000 × *g* for 10 minutes and the final pellet resuspended in isolation medium without EGTA.

#### Particulate Fractions

Particulate fractions were acquired as previously described^23^. Briefly, mouse cortices were washed in cold PBS and then suspended in cell lysis buffer (4 mM ATP, 100 mM KCl, 10 mM imidazole, 2 mM EGTA, 1 mM MgCl_2_, 20% glycerol, 0.05% Triton X-100, 17 g/ml PMSF, 20 g/ml soybean trypsin inhibitor, 25 g/ml leupeptin, and 25 g/ml aprotinin) and homogenized in a glass homogenizer. The homogenate was centrifuged at 1,000 × *g* for 10 minutes and the pellet was resuspended in the cell lysis buffer supplemented with 1% Triton X-100 for 60 minutes. The extracted samples were then centrifuged at 16,000 × *g* for 15 and the supernatant was used as the source of the particulate fraction.

### Sirtuin Activity Assays

Forty-eight hours following ΨεRACK treatment, isolated mitochondria from neuronal-astrocyte cultures (described above) were treated for 45 minutes with 0.1% DMSO or FK866 (50 nM, EMD Millipore), a Nampt inhibitor. Mitochondrial lysine deacetylase activity was analyzed using a SIRT3 activity assay (Cayman Chemical, Ann Arbor, MI). Deacetylase activity was correlated to the fluorescence emitted by the removal of a lysine-acetyl group from a substrate recognized by SIRT3. Similarly, mitochondrial lysine desuccinylase activity was assessed using an assay (BPS Bioscience, San Diego, CA) where the fluorescence emitted from the removal of a lysine-succinyl group from a substrate recognized by SIRT5 correlated to desuccinylase activity. Equal amounts of mitochondrial protein were used across groups.

### Western Blot Analyses

Cells, particulate fraction, or mitochondria were lysed in RIPA buffer pH 8.0 containing 150 mM NaCl, 1% NP-40, 0.5% sodium deoxycholate, 0.1% SDS, 50 mM Tris, supplemented with 1% protease and 1% phosphatase inhibitor cocktails (Sigma) and then centrifuged at 12,000 × g for 15 minutes. Equal amounts of proteins were subjected to 10 to 15% SDS–polyacrylamide gel electrophoresis and the separated proteins were electrophoretically transferred to PVDF membrane (BioRad). The blot was blocked with 5% non-fat dried milk, incubated overnight at 4 °C with SIRT5 (1:1000), SIRT3 (1:1000), acetyl-Lysine (1:500), CoxIV (1:1000), β-actin (1:2000) (all from Cell Signaling Technology, Danvers, MA), PKCε (1:1000), Nampt (1:125) (Santa Cruz Biotechnology), or succinyl-Lysine (1:1000) (PTM BioLabs, Chicago, IL) antibodies then followed by incubation with horseradish peroxidase-conjugated specific secondary antibody (GE Healthcare UK Limited, Little Chalfont, Buckinghamshire) for 1 hour at room temperature. The immunoreactive bands were revealed by ECL western blotting detection reagents (Pierce Thermo Scientific, Rockford, IL). Western blot images were digitized by means of a CCD camera equipped with 50 mm NIKKOR lens (Nikon, Tokyo). The camera was interfaced to the Versadoc Imaging System (BioRad). The digitized immunoblots were subjected to densitometric analysis using Quantity One 1-D Analysis software (BioRad).

### Real-time qPCR

Mice were perfused with cold saline prior to dissection of the brain. Cortices were snap frozen in liquid nitrogen then homogenized in TRIzol (Life Technologies). RNA was extracted using an RNeasy Mini Kit (Qiagen, Hilden, Germany) according to the manufacturer’s instructions. 1 μg of RNA was used as the template for cDNA synthesis by qScript cDNA SuperMix (Quanta Biosciences, Beverly, MA). cDNA was diluted 1:4 prior to real-time qPCR carried out with Power SYBR Green Master Mix (Life Technologies) in triplicate using the LightCycler^®^ 480 II (Roche). Results were analyzed by the ΔΔCT method and presented as fold change of Tat expression. SIRT5 Forward 5′-CCCTTGCTCCTCATGAAACT-3′; SIRT5 Reverse 5′-CCGTTAGTGCCCTGCTTA-3′; β-Actin Forward 5′-CTGTATTCCCCTCCATCGTG-3′; β-Actin Reverse 5′-GGGTCAGGATACCTCTCTTGC-3′.

### Mitochondrial Respiration

Mitochondria isolated from WT or SIRT5−/− homozygous knockout mouse cortex were treated for 45 minutes with 0.1% DMSO, FK866 (50 nM, EMD Millipore), or not treated. Mitochondrial respiration analysis was performed as previously described^7^. In brief, oxygen consumption was measured in a water-jacketed chamber in respiration buffer (composed of 150 mM sucrose, 25 mM Tris-HCl, pH 7.4, and 10 mM potassium phosphate buffer, pH 7.4). Using a Clark-type oxygen electrode, the oxygen signal was acquired using Oxygraph hardware and software (Hansatech Instruments, Norfolk). Mitochondria were normalized based protein concentrations and 50 ug of mitochondria protein of each treatment group was added to the assay. The rate of respiration was measured in the presence of 5 mM pyruvate, 2.5 mM malate, with excess ADP (0.5 mM) and then complex I was inhibited with rotenone (5 mM). Next, 8 mM succinate and 4 mM glycerol-3-phosphate (G3P) were added, then complex III was inhibited with antimycin (10 μM). Finally, 0.5 mM ascorbate and 200 mM *N*, *N*, *N′*, *N′*-tetramethyl-*p*-phenylenediamine (TMPD) were added, then complex IV was inhibited with potassium cyanide (1.8 mM). Metabolites and inhibitors were obtained from Sigma.

### Middle Cerebral Artery Occlusion

WT or SIRT5−/− mice were rapidly anesthetized with 2% isofluorane then maintained on 1% isofluorane in 100% oxygen. A flexible 0.5-mm fiberoptic probe was affixed to the exposed skull to measure blood flow. The right middle cerebral artery was transiently occluded (MCAO) by inserting a silicone-coated 8–0 monofilament nylon surgical suture into the internal carotid artery to the base of the middle cerebral artery. The filament was removed after 85 minutes and the tissue was reperfused. Animals were then placed in an incubator at 37 °C for two hours before being returned to their home cages.

### Infarct Size Determination

Twenty-four hours following MCAO, animals were scored on a neurobehavioral battery based on sensorimotor function as previously described^56^. Mice were then perfused with heparinized saline and brains rapidly removed, placed into a brain matrix, frozen at −80 °C for 7 minutes, and then sliced into 1-mm thick coronal sections. These sections were immersed in 1.5% TTC dissolved in PBS and were incubated in the dark for 10 minutes. The sections were transferred to buffered 10% formalin for fixation. The fixed sections were scanned and infarcts were traced at each level using Image J software (National Institute of Health, Bethesda, MD).

### Statistical Analysis

All data were expressed as the mean ± S.E.M. Statistical significance was determined with Student’s *t*-test for comparison between two groups or one way-analysis of variance (ANOVA) followed by Bonferroni’s multiple comparison test for comparison between more than two groups. In all cases, *p* value less than 0.05 was considered statistically significant.

## Additional Information

**How to cite this article**: Morris-Blanco, K. C. *et al*. Protein Kinase C Epsilon Promotes Cerebral Ischemic Tolerance Via Modulation of Mitochondrial Sirt5. *Sci. Rep*. **6**, 29790; doi: 10.1038/srep29790 (2016).

## Supplementary Material

Supplementary Information
