# Supplementary material for: Protein Kinase C Epsilon Promotes Cerebral Ischemic Tolerance Via Modulation of Mitochondrial Sirt5
Source: Sci Rep. 2016 Jul 20;6:29790. doi: 10.1038/srep29790 (PMC4951704; doi:10.1038/srep29790)
Supplement: Supplementary Information [file srep29790-s1.pdf]

Original Research Communication

PROTEIN KINASE C epsilon PROMOTES CEREBRAL ISCHEMIC TOLERANCE VIA MODULATION OF MITOCHONDRIAL SIRT5

Kahlilia C. Morris-Blanco[1,2,3], Kunjan R. Dave[1,2,3], Isabel Saul[1,3], Kevin B. Koronowski[1,2,3], Holly M. Stradecki[1,2,3], & Miguel A. Perez-Pinzon*[1,2,3]

Cerebral Vascular Disease Research Laboratories[1], Neuroscience Program[2], Department of Neurology[3], University of Miami Miller School of Medicine, Miami, FL 33136

Supplementary Figure S1

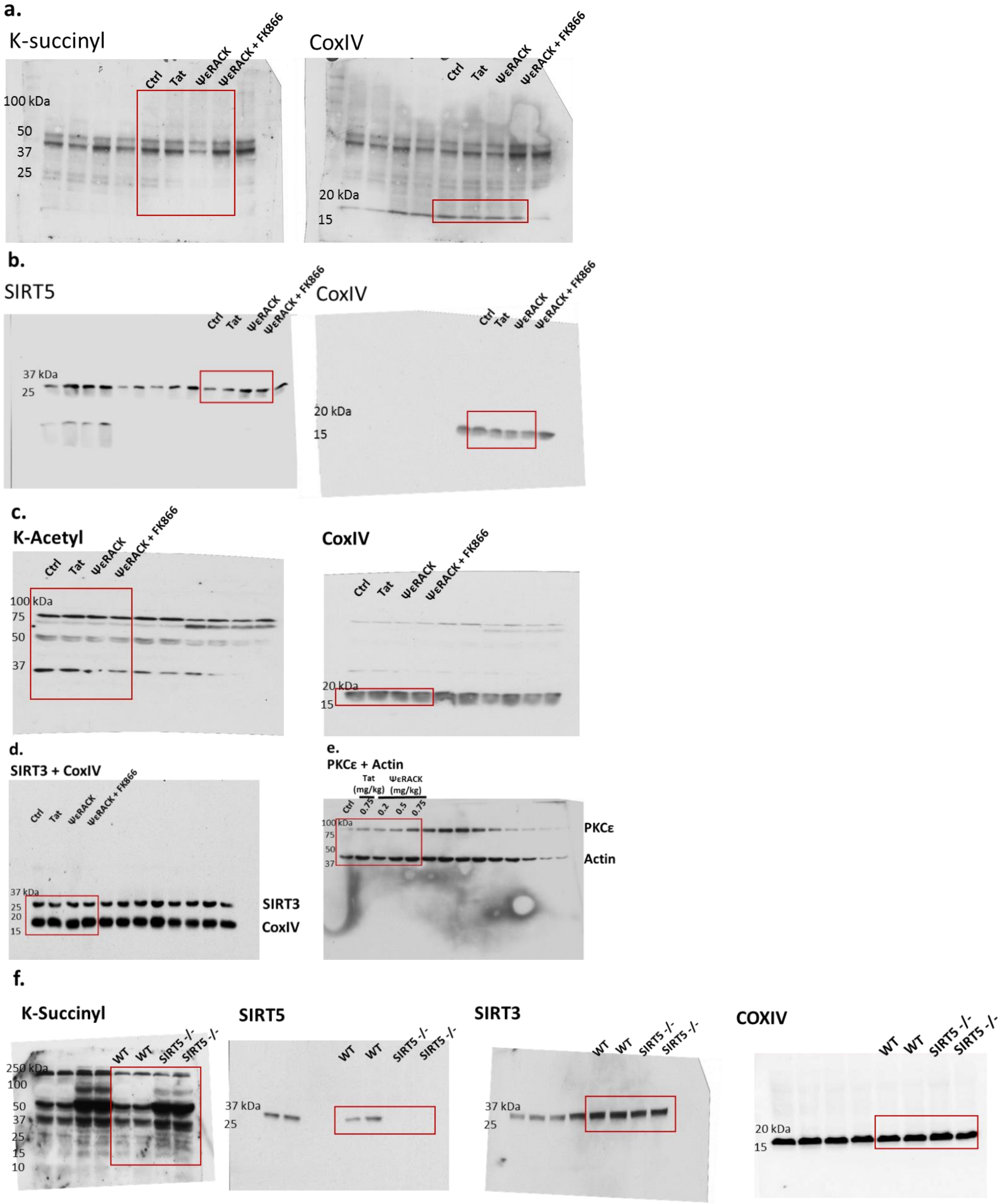

Supplementary Figure S1. Full-length Western Blot Images. a,b) Figure 2 blots. c,d) Figure 3 blots. e) Figure 4 blots. f) Figure 5 blots.
